# Use of Nonsteroidal Anti-Inflammatory Drugs and Bladder Cancer Risk: A Meta-Analysis of Epidemiologic Studies

**DOI:** 10.1371/journal.pone.0070008

**Published:** 2013-07-19

**Authors:** Haifeng Zhang, Dongpeng Jiang, Xuedong Li

**Affiliations:** 1 Department of Urology Surgery, The Second Clinical College, Harbin Medical University, Harbin, Heilongjiang Province, China; 2 Department of Urology Surgery, Heilongjiang Provincial Corps Hospital of the Chinese People’s Armed Police Force, Harbin, Heilongjiang Province, China; The University of Texas M. D. Anderson Cancer Center, United States of America

## Abstract

**Purpose:**

Several epidemiologic studies have evaluated the association between nonsteroidal anti-inflammatory drugs (NSAIDs) and bladder cancer risk and the results were varied. Thus, we conducted a comprehensive meta-analysis of studies exclusively dedicated to the relationship between the 3 most commonly used analgesics and bladder cancer risk.

**Methods:**

A systematic literature search up to November 2012 was performed in PubMed database for 3 categories of analgesics: acetaminophen, aspirin or non-aspirin NSAIDs. Study-specific risk estimates were pooled using a random-effects model.

**Results:**

Seventeen studies (8 cohort and 9 case-control studies), involving a total of 10,618 bladder cancer cases, were contributed to the analysis. We found that acetaminophen (relative risk [RR] 1.01, 95% confidence interval [CI] 0.88–1.17) and aspirin (RR 1.02, 95% CI 0.91–1.14) were not associated with bladder cancer risk. Although non-aspirin NSAIDs was statistically significantly associated with reduced risk of bladder cancer among case-control studies (but not cohort studies), the overall risk was not statistically significant (RR 0.87, 95% CI 0.73–1.05). Furthermore, we also found that non-aspirin NSAIDs use was significantly associated with a 43% reduction in bladder cancer risk among nonsmokers (RR 0.57, 95% CI 0.43–0.76), but not among current smokers.

**Conclusion:**

The results of our meta-analysis suggest that there is no association between use of acetaminophen, aspirin or non-aspirin NSAIDs and bladder cancer risk. However, non-aspirin NSAIDs use might be associated with a reduction in risk of bladder cancer for nonsmokers.

## Introduction

Bladder cancer is the most common malignant tumor of the urinary system. According to the International Agency for Research on Cancer for 2008, about 386,300 individuals were diagnosed with bladder cancer and 150,200 died as a result. The majority of bladder cancer occurs in males and the highest incidence rates are found in the countries of Europe, North America, and Northern Africa [1]. In the US, bladder cancer is the fourth most common cause of cancer among men and the ninth most common cause of cancer death among men [2]. The overall public health importance of bladder cancer is increasing with the growing elderly population.

Cigarette smoking and occupational exposures are the main risk factors for bladder cancer in Western countries, whereas chronic infection with Schistosoma hematobium in developing countries accounts for about 50% of the total burden [3]. Other environmental factors, including selenium intake [4], chlorination by-products [5] and low dose arsenic levels in drinking water [6], have also been associated with bladder cancer, but are less well-established.

Nonsteroidal anti-inflammatory drugs (NSAIDs) are among the most frequently used drugs worldwide. Experimental and epidemiologic evidence strongly suggests that aspirin and non-aspirin NSAIDs have shown promise as chemopreventive agents [7]. Most epidemiologic studies have reported inverse associations between NSAIDs use and the risk of breast [8], gastric [9], and colorectal cancer [10]. However, whether NSAIDs use may reduce the risk of bladder cancer remains unclear. There have been few meta-analyses of NSAIDs use and cancer risk in general, which included some studies of bladder cancer and did not exclusively focus on this disease [11]. The effect of NSAIDs on the risk of bladder cancer remains to be determined. Therefore, we conducted a comprehensive meta-analysis of studies exclusively dedicated to the relationship between the 3 most commonly used analgesics and bladder cancer risk.

## Materials and Methods

### Search Strategy

A systematic literature search up to November 1 of 2012 was performed in PubMed database to identify eligible studies. Search terms included “acetaminophen,” “aspirin,” “nonsteroidal anti-inflammatory agents,” or “NSAID” combined with “bladder cancer,” “bladder neoplasms,” or “bladder carcinoma”. The titles and abstracts of the studies identified in the search were scanned to exclude any clearly irrelevant studies. The full texts of the remaining articles were read to determine whether they contained information on the topic of interest. Furthermore, we also manually searched the reference lists of every article retrieved and review papers to find any additional published studies. All searches were conducted independently by 2 authors (HZ and DJ). The results were compared, and any questions or discrepancies were resolved through iteration and consensus.

### Study Selection

To be eligible, studies had to fulfill the following 4 inclusion criteria: 1) had a case-control or prospective study design; 2) reported results on aspirin, non-aspirin NSAIDs or acetaminophen use; 3) the outcome was bladder cancer incidence or mortality; and 4) reported the estimate of relative risk (RR) with their corresponding 95% confidence interval (CI) (or sufficient data to calculate of these effect measure). Studies reporting different measures of RR like risk ratio, rate ratio, hazard ratio (HR), and odds ratio (OR) were included in the meta-analysis. In practice, these measures of effect yield a similar estimate of RR, since the absolute risk of bladder cancer is low.

### Data Extraction

Data abstraction was conducted independently by 2 researchers (HZ and DJ), with disagreements resolved by consensus. The following information were collected: the first author’s last name, year of publication, country in which the study was performed, study design, years of follow-up or the study period, study participants age range, number of subjects and number of bladder cancer cases, used drugs, exposure definition, information source, control of confounding factors by matching or adjustment, and RR estimates with corresponding 95% CIs. If a study provided several risk estimates, the most completely adjusted estimate was extracted. Differences in data extraction were resolved by consensus, referring back to the original article.

### Statistical analysis

Separate analyses were performed according to use of acetaminophen, aspirin, and non-aspirin NSAIDs. Study-specific risk estimates were extracted from each article, and log risk estimates were weighted by the inverse of their variances to obtain a pooled risk estimate. We pooled study-specific log RRs to compute an overall RR and its 95% CI for regular/any use versus reference group from each study. For reference group, it was defined as “subjects who never took analgesics or who were not regular takers”. Where data for different intake levels or different duration of use were available, we subsequently restricted the analyses to the highest intake or the longest duration given by each study. Studies were combined by using the DerSimonian and Laird random-effects model, which considers both within- and between-study variations [12].

Statistical heterogeneity among studies was assessed using the Cochrane’s Q statistic, and inconsistency was quantified with the *I^2^* statistic that estimates the percentage of total variation across studies due to heterogeneity rather than chance [13]. For the Q statistic, a *P* value <0.10 was considered statistically significant for heterogeneity; for *I^2^*, a value >50% is considered a measure of severe heterogeneity. When statistical heterogeneity was detected, sensitivity analyses were performed. Publication bias was evaluated with Egger’s regression test in which *P* value less than 0.10 was considered representative of statistically significant publication bias [14]. All statistical analyses were performed with Stata 10 software (Stata Corporation, College Station, Texas). We performed this meta-analysis in accordance with the guidelines of the Preferred Reporting Items for Systematic Reviews and Meta-analyses (PRISMA) statement [15].

## Results

### Literature Search

**Figure 1:**
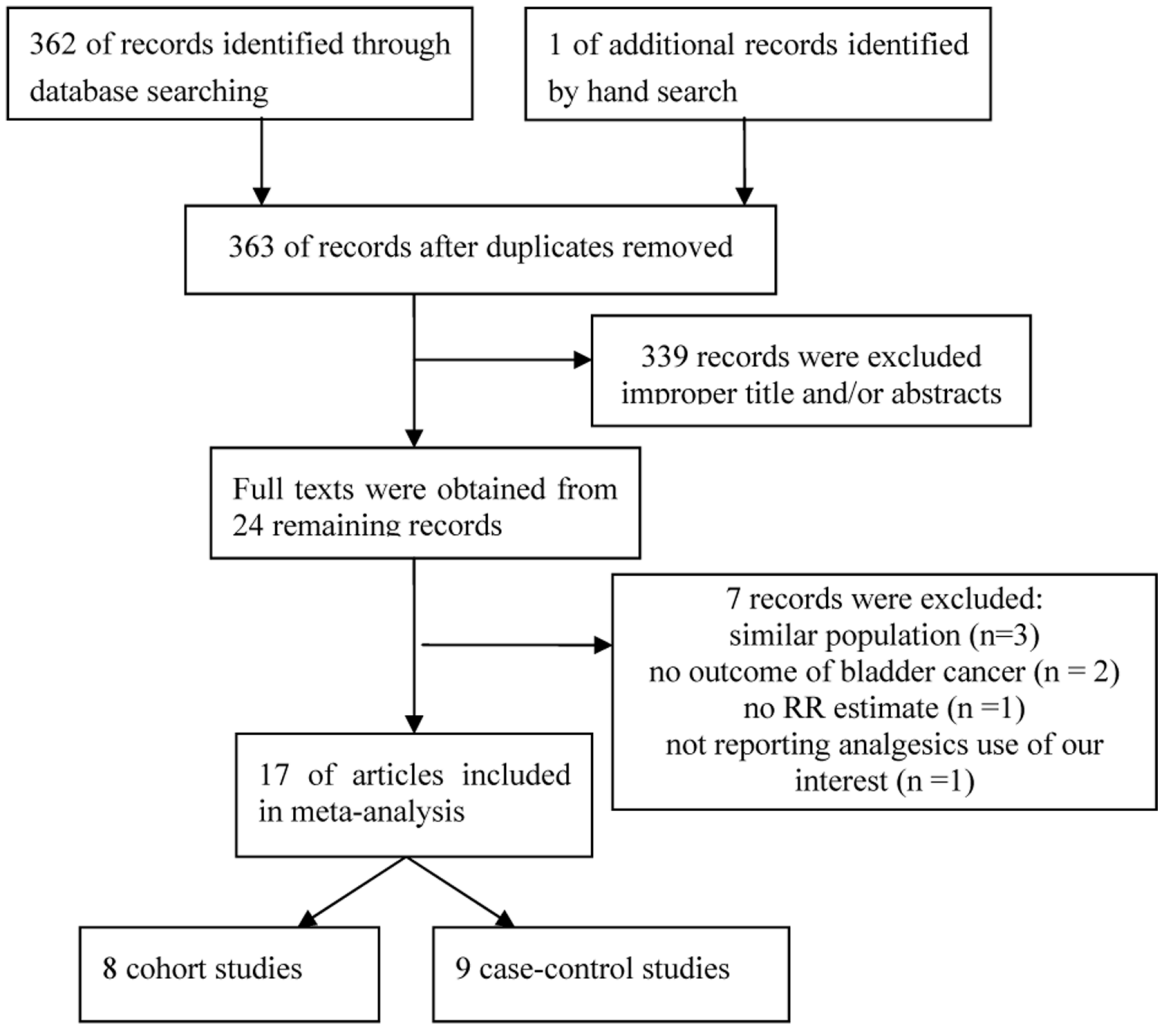
Flow diagram of study identification.

The detailed steps of our literature search are shown in Figure 1. Briefly, our initial search strategy retrieved a total of 363 citations. After the titles and abstracts were screened, 339 articles were excluded because they were laboratory studies, review articles, or irrelevant to the current study. We identified 24 potentially relevant articles. Three articles were excluded because they reported on similar population [16]–[18]. Two publications were excluded because there were no outcomes of bladder cancer [19], [20], one was excluded because it did not provide RR estimate [21] and the remaining one was excluded because it did not report analgesics use of our interest [22]. Finally, 17 articles [23]–[39] were included in this meta-analysis (Figure 1).

The 17 relevant studies were published between 1985 and 2012, including 8 cohort studies [28]–[31], [35]–[38] and 9 case-control studies [23]–[27], [32]–[34], [39]. A total of 1,008,800 participants, including 10,618 bladder cancer cases were involved in these studies and followed for 3–21 years. Ten studies were used for analysis of acetaminophen use [23]–[28], [33]–[35], [39], 11 for aspirin use [25], [26], [29], [31], [33]–[39] and 6 for non-aspirin NSAIDs use [30], [32], [33], [36], [38], [39]. The characteristics of the included studies for the 3 most commonly used analgesics are summarized in Table 1. Most studies provided risk estimates that were adjusted for age (16 studies), sex (14 studies) and smoking (12 studies); fewer were adjusted for race (6 studies), body mass index (4 studies), and education (4 studies). The exposure definitions of the included studies are shown in Table 2.

**Table 1:** Characteristics of studies included in the meta-analysis.

Study	Year	Country	Design	Studyperiod	Age, y	N. ofparticipant	BC Cases	Drug(s)	Informationsource	Adjustments*
Piper (23)	1985	USA	C-C	1975–1979	20–49	173	173	ACE	Interview	1–3
Derby (24)	1996	USA	C-C	1980–1991	≥20	2,009	504	ACE	Database	1, 2, 4, 5
Pommer (25)	1999	Germany	C-C	1990–1994	70.4	921	571	ACE/ACA	Interview	1, 6–8
Castelao (26)	2000	USA	C-C	1987–1996	25–74	1514	1514	ACE/ACA	Interview	1, 2, 6, 9, 10
Kaye (27)	2001	USA	C-C	1995–1998	>50	187	744	ACE	Database	1, 2, 6, 11, 12, 13, 14
Friis (28)	2002	Denmark	Co	1989–1995	63	39,946	115	ACE	Database	1, 2
Friis (29)	2003	Denmark	Co	1989–1997	70	29,470	161	ASA	Database	1, 2
Sørensen (30)	2003	Denmark	Co	1989–1995	47.2	172,057	330	NA-NSAIDs	Database	1, 2
Ratnasinghe (31)	2004	USA	Co	1971–1992	25–74	22,843	40	ASA	Interview	2, 6,7, 9, 14, 15,
Blumentals (32)	2004	USA	C-C	1992–1994	71.1	1,293	330	NA-NSAIDs	Database	1, 2, 6, 16
Fortuny(33)	2006	Spain	C-C	1997–2000	20–80	1,029	958	ACE/ACA/NA-NSAIDs	Interview	1, 2, 3, 6, 16
Fortuny (34)	2007	USA	C-C	1998–2001	25–74	463	376	ACE/ACA	Interview	1, 2, 6, 16
Genkinger (35)	2007	USA	Co	1986–2004	40–75	49,448	607	ACE/ACA	Questionnaire	1, 3, 6, 17, 18
Daugherty (36)	2011	USA	Co	1993–2005	62.1	508,842	2,489	ASA/NA-NSAIDs	Questionnaire	6, 14–16, 19
Jacobs (37)	2012	USA	Co	1997–2008	NR	100,139	150	ASA	Questionnaire	1, 2, 6, 9, 14–16, 20– 26
Shih (38)	2012	USA	Co	2000–2010	50–76	77,048	385	ASA/NA-NSAIDs	Questionnaire	1, 2, 6, 9, 15, 16, 27
Baris (39)	2013	USA	C-C	2001–2004	30–79	1,418	1,171	ACE/ACA/NA-NSAIDs	Interview	1, 2, 3, 6, 15, 28

Abbreviations: BC, bladder cancer; C-C, case control; Co, cohort; NR, not reported; ACE: Acetaminophen; ASA: aspirin; NA-NSAIDs: nonaspirin NSAIDs.

* 1, age; 2, sex; 3, residence; 4, certain occupations; 5, coffee drinking; 6, smoking; 7, socioeconomic status; 8, laxative intake, 9, education; 10, number of years employed as hairdresser/barber, 11, general practice; 12, duration of prescription history in the database; 13, index date; 14, body mass index, 15, race; 16, analgesic use; 17, period;18, fluid intake; 19, study; 20, physical activity; 21, history of heart disease; 22, stroke; 23, diabetes; 24, hypertension; 25, cholesterol-lowering drug use; 26, history of colorectal endoscopy; 27, family history of bladder cancer; 28, Hispanic status.

**Table 2:** Exposure definition in each study.

Study	Exposure definition
Piper (23)	Regular use (daily use for at least 30 days per year) vs. no use
Derby (24)	Any use (≥1 prescription in past year) vs. no use
Pommer (25)	Regular use (lifelong cumulative amount of ≥1 kg) vs. no use
Castelao (26)	Regular use (≥2 times a week for ≥1 month) vs. no/irregular use
Kaye (27)	Any use (≥1 prescription) vs. no use
Friis (28)	Any use(≥1 prescription) vs. no use
Friis (29)	Regular use low-dose aspirin (75–150 mg once daily) vs. no use
Sørensen (30)	Any use (≥1 prescription) vs. no use
Ratnasinghe (31)	Any use (use any aspirin in past 30 days or 6 months ) vs. no use
Blumentals (32)	Any use (≥1 prescription) vs. no use
Fortuny(33)	Regular use (≥2 times a week for ≥1 month) vs. no use
Fortuny (34)	Regular use (≥4 times a week for ≥1 month) vs. no use
Genkinger (35)	Regular use (≥2 times a week) vs. no use
Daugherty (36)	Regular use (>2 times a week) vs. no use
Jacobs (37)	Regular use (daily use) vs. no use
Shih (38)	Regular use (>1 tine a week for ≥1 year) vs. no use
Baris (39)	Regular use (≥2 times a week for ≥1 month) vs. no use

### Acetaminophen

**Figure 2:**
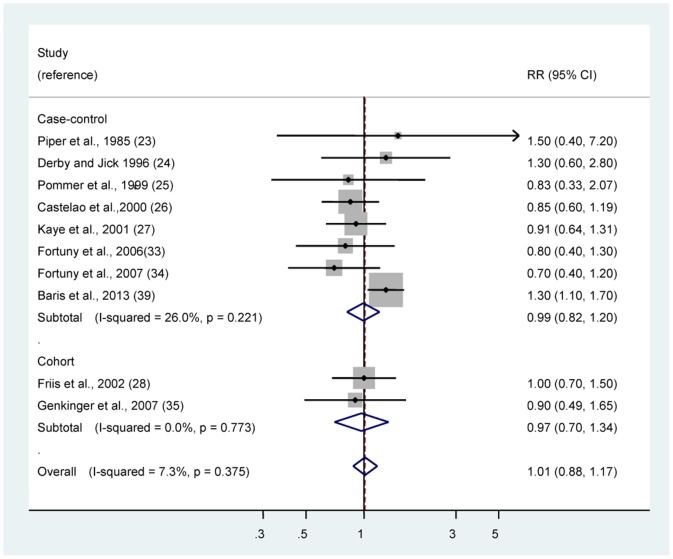
Risk estimates of bladder cancer associated with regular/any use of acetaminophen. Squares indicate study-specific risk estimates (size of the square reflects the study-specific statistical weight, i.e., the inverse of the variance); horizontal lines indicate 95% confidence intervals (CIs); diamonds indicate summary risk estimate with its corresponding 95% confidence interval.

The multivariable-adjusted RRs of bladder cancer for regular/any use of acetaminophen in individual observational studies and summary estimate are shown in Figure 2. Regular/any use of acetaminophen was not associated with the risk of bladder cancer (RR 1.01, 95% CI 0.88–1.17). The Cochran’s Q test resulted in a *P* = 0.38 (Q = 9.70), and the corresponding quantity *I^2^* was 7.3%, both indicating that the results of those studies were homogeneous. The *P* value for the Egger test was *P* = 0.23, suggesting a low probability of publication bias. The associations of acetaminophen use with bladder cancer risk did not differ by study type (Table 3).

**Table 3:** Summary risk estimates.

Stratification group	References	RR (95% CI)		Heterogeneity test		
				*Q*	*P*	*I^2^* (%)†
Acetaminophen						
Regular/any use	23–28, 33–35, 39	1.01	0.88–1.17	9.70	0.375	7.3
Case-control studies	23–27, 33, 34, 39	0.99	0.82–1.20	9.46	0.221	26.0
Cohort studies	28, 35	0.97	0.70–1.34	0.08	0.773	0
High intake	24, 26–28, 33, 39	1.08	0.81–1.44	3.98	0.552	0
Long duration	33, 34, 39	0.80	0.43–1.48	3.53	0.171	43.4
US	23, 24, 26, 27, 34, 35, 39	1.01	0.82–1.23	8.63	0.195	30.5
Europe	25,28,33	0.92	0.68–1.25	0.45	0.800	0
Aspirin						
Regular/any use	25, 26, 29, 31, 33–39	1.02	0.91–1.14	19.49	0.035	48.7
Case-control studies	25, 26, 33, 34, 39	0.95	0.76–1.18	9.94	0.042	59.7
Cohort studies	29, 31, 35–38	1.06	0.93–1.20	8.51	0.130	41.2
High intake	26, 33, 35, 36, 38, 39	0.96	0.83–1.12	7.04	0.218	29.0
Long duration	33, 34, 37, 39	1.04	0.82–1.32	0.93	0.819	0
Men	29,31,36	1.10	0.99–1.21	2.08	0.354	3.7
Women	29,31,36	1.43	0.68–3.00	13.12	0.001	84.8
No smoker	35, 36, 38	0.90	0.75–1.09	0.24	0.885	0
Current smoker	35, 36, 38	0.99	0.81–1.21	0.04	0.982	0
US	26, 31, 34–39	0.97	0.84–1.12	16.06	0.025	56.4
Europe	25, 29, 33	1.16	0.99–1.33	0.83	0.662	0
Non-aspirin NSAIDs						
Regular/any use	30, 32, 33, 36, 38, 39	0.87	0.73–1.05	24.19	<0.001	79.3
Case–control studies	32, 33,39	0.76	0.62–0.95	3.19	0.203	37.3
Cohort studies	30, 36, 38	0.98	0.78–1.22	11.87	0.003	83.1
No smoker	32, 36, 38	0.57	0.43–0.76	0.04	0.979	0
Current smoker	36, 38	1.24	0.63–2.46	2.31	0.129	56.7
US	32, 36, 38, 39	0.86	0.79–0.94	2.19	0.534	0
Europe	30, 33	0.74	0.25–2.15	7.96	0.005	87.4

Abbreviation: RR, relative risk; CI, confidence intervals.

† *I^2^* is interpreted as the proportion of total variation across studies that are due to heterogeneity rather than chance.

Further, six studies [24], [26]–[28], [33], [39] were reported RR estimates of the association between high intake of acetaminophen and bladder cancer risk (Table 3). Based on the results from those studies, the calculated combined RR for bladder cancer in high intake of acetaminophen was found to be 1.08 (95% CI 0.81–1.44). And the association was also not significant (RR 0.80, 95% CI 0.43–1.48) with long duration of acetaminophen use among limited number of studies with that available information [33], [34], [39]. Stratified analysis by country did not show any statistically significant difference in summary estimates between strata.

### Aspirin

**Figure 3:**
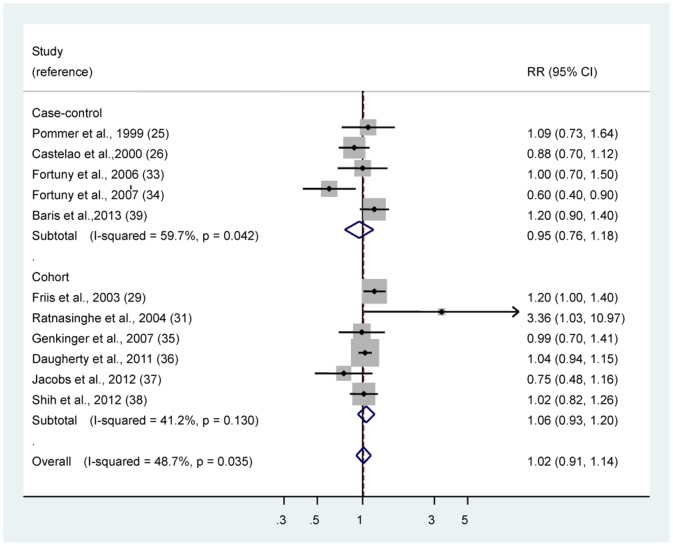
Risk estimates of bladder cancer associated with regular/any use of aspirin. Squares indicate study-specific risk estimates (size of the square reflects the study-specific statistical weight, i.e., the inverse of the variance); horizontal lines indicate 95% confidence intervals (CIs); diamonds indicate summary risk estimate with its corresponding 95% confidence interval.

The multivariable-adjusted RRs of bladder cancer for regular/any use of aspirin in individual observational studies and summary estimate are shown in Figure 3. The pooled RR of bladder cancer for regular/any aspirin use was 1.02 (95% CI 0.91–1.14). There was statistically significant heterogeneity among studies (*P = *0.035, *I^2^* = 48.7%). The Egger test showed no evidence of publication bias for aspirin (*P = *0.686). The associations of aspirin use with bladder cancer risk did not differ by study type (Table 3).

To explore the heterogeneity among studies of aspirin use and bladder cancer, we performed sensitivity analyses. By using a stepwise process, we determined that most of heterogeneity was accounted for the study by Fortuny et al [34]. After excluding this single study, there was no study heterogeneity (*P = *0.20, *I^2^* = 26.4%), and the RR was essentially unchanged (RR 1.05, 95% CI 0.96–1.15).

High intake or long duration of aspirin use was also not associated with bladder cancer risk (Table 3). In addition, stratified analysis by smoking status, gender and country did not show any statistically significant difference in summary estimates between strata.

### Non-aspirin NSAIDs

**Figure 4:**
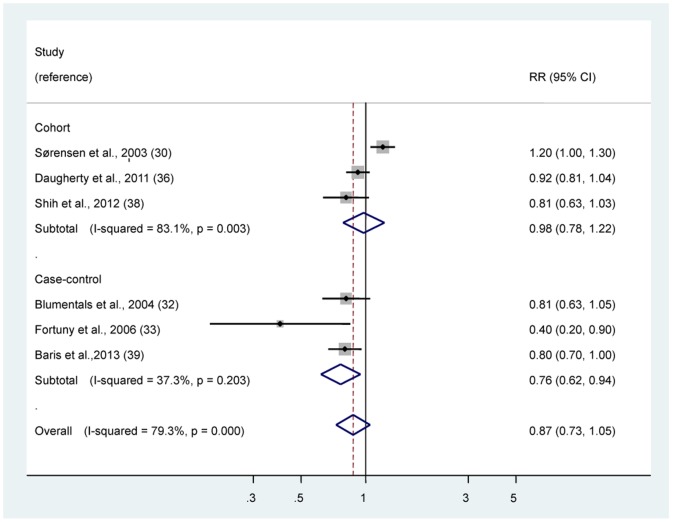
Risk estimates of bladder cancer associated with regular/any use of non-aspirin NSAIDs. Squares indicate study-specific risk estimates (size of the square reflects the study-specific statistical weight, i.e., the inverse of the variance); horizontal lines indicate 95% confidence intervals (CIs); diamonds indicate summary risk estimate with its corresponding 95% confidence interval.

Six studies [30], [32], [33], [36], [38], [39] were reported RR estimates of the association between non-aspirin NSAIDs use and bladder cancer risk (Figure 4). No association was observed between regular/any non-aspirin NSAIDs use and the risk of bladder cancer (RR 0.87, 95% CI 0.73–1.05). There was statistically significant heterogeneity among studies (*P*<0.001, *I^2^* = 79.3%). The Egger test showed no evidence of publication bias for aspirin (*P = *0.118). And we found that non-aspirin NSAIDs use was statistically significantly associated with reduced risk of bladder cancer among case-control studies, but not among cohort studies (Table 3).

To explore the heterogeneity, we also performed the sensitivity analyses. By using a stepwise process, we determined that most of heterogeneity was accounted for the study by Sørensen et al [30]. After excluding this single study, there was no study heterogeneity (*P = *0.19, *I^2^* = 34.7%), and a significant association was observed (RR 0.83, 95% CI 0.74–0.94).

In addition, stratified analysis by smoking status found that non-aspirin NSAIDs use was statistically significantly associated with 43% reduced risks of bladder cancer among nonsmokers (RR 0.57; 95% CI, 0.43–0.76), but not among current smokers (RR 1.24; 95% CI, 0.63–2.46) (Table 3). And we found that non-aspirin NSAIDs use was statistically significantly associated with reduced risk of bladder cancer among studies from the US, but not among studies from Europe.

## Discussion

This present meta-analysis included 17 studies (8 cohort and 9 case-control studies), involving a total of 10,618 bladder cancer cases. In this meta-analysis of the 3 most commonly used analgesics and bladder cancer risk, we found that acetaminophen and aspirin were not associated with bladder cancer risk. Although non-aspirin NSAIDs was statistically significantly associated with reduced risk of bladder cancer among case-control studies (but not cohort studies), the overall risk was not statistically significant. Also, non-aspirin NSAIDs was significantly associated with a 43% reduction in bladder cancer risk among nonsmokers.

We found that acetaminophen use was not associated with the risk of bladder cancer with a pooled RR = 1.01 from 10 studies. Acetaminophen is a metabolite of phenacetin, a well-known banned carcinogen which has been more so linked to renal pelvic tumor [40]. Recently, a meta-analysis found that acetaminophen use was associated with 21% reduced risk of kidney cancer and the risk was higher with higher intake. In this meta-analysis, we did not found the associations of long term and high dose of acetaminophen use with bladder cancer risk.

In our meta-analysis, we did not found that aspirin use was associated with bladder cancer risk from 11 studies. Our results are in agreement with a large randomized controlled trial [41]. In the Women’s Health Study, low dose aspirin (100 mg every other day) for an average of ten years did not lower bladder cancer incidence. And in the meta-analysis of aspirin and cancer risk, the pooled risk for bladder cancer was 0.95 (95% CI 0.83–1.07) [11].

We found from 6 studies that non-aspirin NSAIDs use was not associated with the risk of bladder cancer in general. Nevertheless, there was significant heterogeneity by study. After excluding the study by Sørensen et al [30], a significant inverse association was found. In this record linkage study from Denmark with 330 bladder cancer cases, prescribed non-aspirin NSAIDs slightly increased the risk of bladder cancer (RR 1.2; 95% CI, 1.0–1.3), although no dose-response relationship was observed. Misclassification is likely in studies relying solely on prescription data as many commonly used NSAIDs do not require a prescription. Therefore, due to the limited published information and relatively small number of cases, we still cannot draw the firm conclusion about non-aspirin NSAIDs use and bladder cancer risk. Further studies with a larger number of subjects may be able to discriminate the effects of specific NSAIDs more clearly.

Furthermore, an interest finding in our meta-analysis, although more limited, is that smoking status might modify the association of use of nonaspirin NSAIDs with bladder cancer risk. Smoking is a well-known risk factor for bladder cancer. There are over 60 carcinogens, including nitrosamines, aromatic amines, and polycyclic aromatic hydrocarbons, have been detected in cigarette smoke. Metabolic activation of these carcinogens leads to the formation of DNA adducts, causing miscoding and other mutations [42]. While smokers have been shown to have increased cycloxygenase (COX)-2 expression and activity in their urothelial tissues [43], it may be that the anticarcinogenic effects of nonaspirin NSAIDs against COX-2 are overwhelmed by the carcinogenic effects of smoking. Thus, a decrease in bladder cancer risk associated with nonaspirin NSAIDs has been shown in nonsmokers in our meta-analysis.

Our study has several strengths: it is the most up-to-date comprehensive review of analgesics on one specific type of cancer, bladder cancer. It includes the 3 mostly used contemporary drugs and 17 observational studies were included in our meta-analysis, reporting data of more than 1 million participants, including 10,618 bladder cancer patients. Meta-analysis of studies with large numbers of incident cases provides high statistical power for estimating the relationship between exposure and outcome risk. Moreover, in a meta-analysis of published studies, publication bias could be of concern since small studies with null results tend not to be published. In this meta-analysis, however, we found little evidence of publication bias.

Nevertheless, several limitations are worth mentioning. First, a meta-analysis is not able to solve problems with confounding factors that could be inherent in the included studies. Inadequate control for confounders may bias the results in either direction, toward exaggeration or underestimation of risk estimates. However, most observational studies in this meta-analysis adjusted for other known and potential risk factors for bladder cancer. Second, heterogeneity may be introduced because of methodologic and demographic differences among studies. We used appropriate well-motivated inclusion criteria to maximize homogeneity, and performed sensitivity and subgroup analyses to investigate potential sources of heterogeneity. Third, studies used different definition of analgesic use, which might have limited comparability of the results across the studies. However, our findings were stable and robust in the subgroup analyses. Fourth, long-term analgesic users may switch the type of analgesics they use over time. Because almost all of the studies assessed analgesics use at baseline only, we were not able to evaluate the impact of change of use of analgesics. In addition, the study does not clearly distinguish between multiple exposures to analgesics. The possibility of confounding would be especially relevant for long duration users, as those patients could have used phenacetin many years previously. Last, because of lack of data, it was not possible to address the important issues of dose and duration of use needed to achieve effects.

In summary, the results of this meta-analysis of 17 observational studies indicated that use of acetaminophen, aspirin or non-aspirin NSAIDs was not associated with bladder cancer risk. However, non-aspirin NSAIDs use might be associated with a reduction in risk of bladder cancer for nonsmokers.

## Supporting Information

Checklist S1 **PRISMA Checklist for the meta-analysis.**
(DOC)
